# Supplementary material for: Endothelial Progenitor Cells Promote Osteosarcoma Progression and Invasiveness via AKT/PI3K Signaling
Source: Cancers (Basel). 2023 Mar 17;15(6):1818. doi: 10.3390/cancers15061818 (PMC10046883; doi:10.3390/cancers15061818)
Supplement: Supplementary file 1 [file cancers-15-01818-s001.zip › cancers-2122940-supplementary.pdf]

Endothelial Progenitor Cells Promote Osteosarcoma Progression and Invasiveness via AKT/PI3K Signaling

Ofri Doppelt-Flikshtain [1,2], Amin Younis [1,2,3], Tal Tamari [1,2], Ofir Ginesin [1,2,3], Talia Shentzer-Kutiel [4], David Nikomarov [5], Gil Bar-Sela [2,6], Benjamin R. Coyac [1,3], Yehuda G. Assaraf [7] and Hadar Zigdon-Giladi [1,2,3,*]

[1] Laboratory for Bone Repair, Rambam Health Care Campus, Haifa 3109601, Israel
[2] The Ruth and Bruce Rappaport Faculty of Medicine, Technion-Israel Institute of Technology, Haifa 3525422, Israel
[3] Department of Periodontology, School of Graduate Dentistry, Rambam Health Care Campus, Haifa 3109601, Israel
[4] Thoracic Cancer Service, Rambam Health Campus, Haifa 3109601, Israel
[5] Musculoskeletal Oncology Department, Rambam Health Campus, Haifa 3109601, Israel
[6] Oncology Departmant, Emek Medical Center, Afula 1834111, Israel
[7] The Fred Wyszkowski Cancer Research Laboratory, Department of Biology, Technion-Israel Institute of Technology, Haifa 3200003, Israel

***Corresponding Author:** Prof. Hadar Zigdon-Giladi, Research Institute for Bone Repair and the Department of Periodontology, School of Graduate Dentistry, Rambam Health Care Campus, P.O. Box 9602, Haifa, 31096, Israel | Tel. +972-4-8543606, Fax. +972-4-8542467 | Email: hadar@technion.ac.il

Characterization of endothelial progenitor cells

Characterization of late EPCs at passages 3–5 isolated from peripheral blood of healthy donors was performed by flow cytometry FACS analysis. Colonies of adherent proliferating cells with cobble stone morphology appeared in the culture 2–3 weeks after seeding with circulating mononuclear cells. According to flow cytometry analysis, high percentages of endothelial progenitor markers were expressed in late EPCs: CD31 (97.7 ± 3.1%), VEGFR-2 (69.5 ± 36.7%), and CD34 (81.2 ± 23.5%). Whereas low percentages of the monocyte/macrophage marker CD14 (4.1 ± 4.7%) and hematopoietic marker CD45 (10.4 ± 6.5%) were found. Endothelial progenitor cells were also characterized by cell morphology as well as by functional assays. In addition, non-proliferating early EPCs appear early (4-7 days) in culture dishes, compared to late EPCs that appear late (2–4 weeks after seeding) and form colonies of proliferative cells with a cobblestone-like morphology.

Real Time PCR primer sequence:

Primer sequences: MMP9 Forward: 5′ TTGACAGCGACAAGAAGTGG -3′ ; Reverse: 5′ -GCCATTCACGTCGTCCTTAT-3′ . As an internal control, the levels of Hypoxanthine phosphoribosyltransferase (HPRT) and Glyceraldehyde-3-Phosphate Dehydrogenase GAPDH were quantified in parallel with the above target genes. Primer sequences: HPRT- Forward: 5'-ATGACAGCTGCACCACTGAG-3'; Reverse: 5'-ATTTGTTGCCCAGGAAAGTG-3'. GAPDH- Forward: 5′ -TTGACAGCGACAAGAAGTGG-3′ ; Reverse: 5′ -GCCATTCACGTCGTCCTTAT-3′ .

Table S1: IHC antibody and cases included for each antibody.

Human			Mice	
	Primary tumor	Metastasis		Primary tumor
CD31	7	18	X	X
VEGF-A	7	8	VEGF-A	5
FGF2	6	14	FGF2	5

Immunohistochemistry analysis- negative control

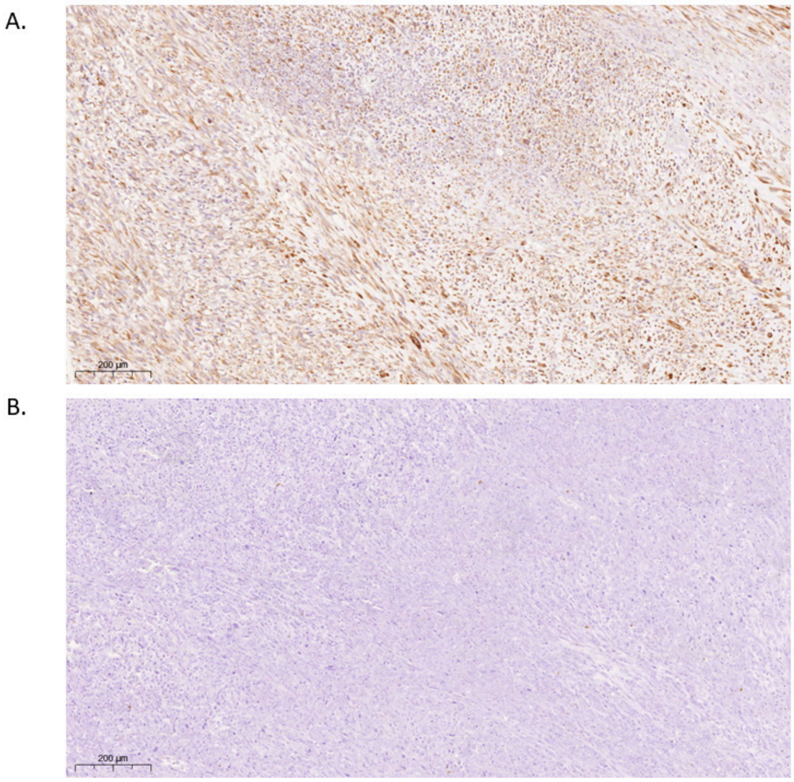

Figure S1-1: Representative image of a negative control. Slides were labeled with and without primary antibody. A. VEGF-A labeling. B. Negative control, without primary antibody. Images were obtained under x10 microscope magnification. Scale bar denotes 200 µm.

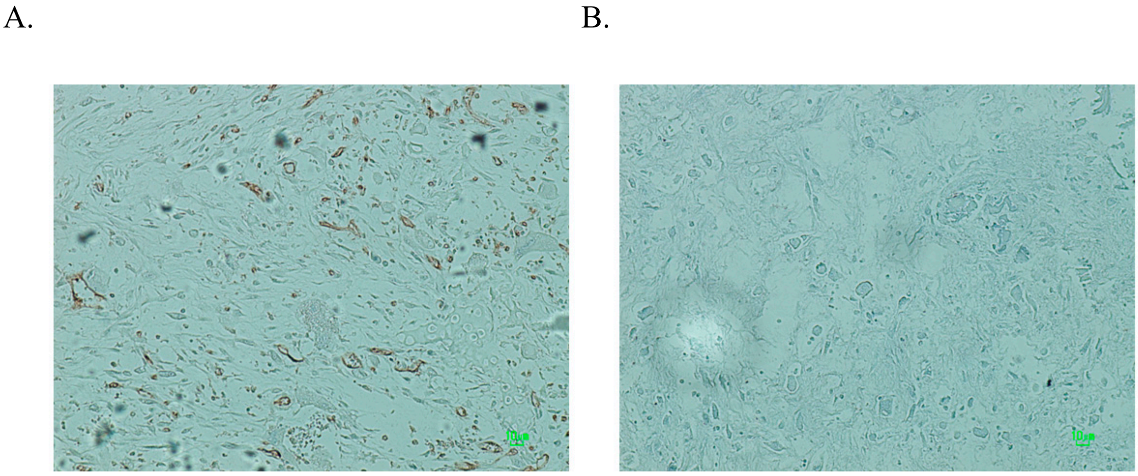

Figure S1-2: Representative image of a negative control. Slides were labeled with and without primary antibody. A. CD31 labeling. B. Negative control, without primary antibody. Images were obtained under x10 microscope magnification. Scale bar denotes 10 µm.

EPCs promote 143B cell migration and invasion via PI3K/AKT signaling.

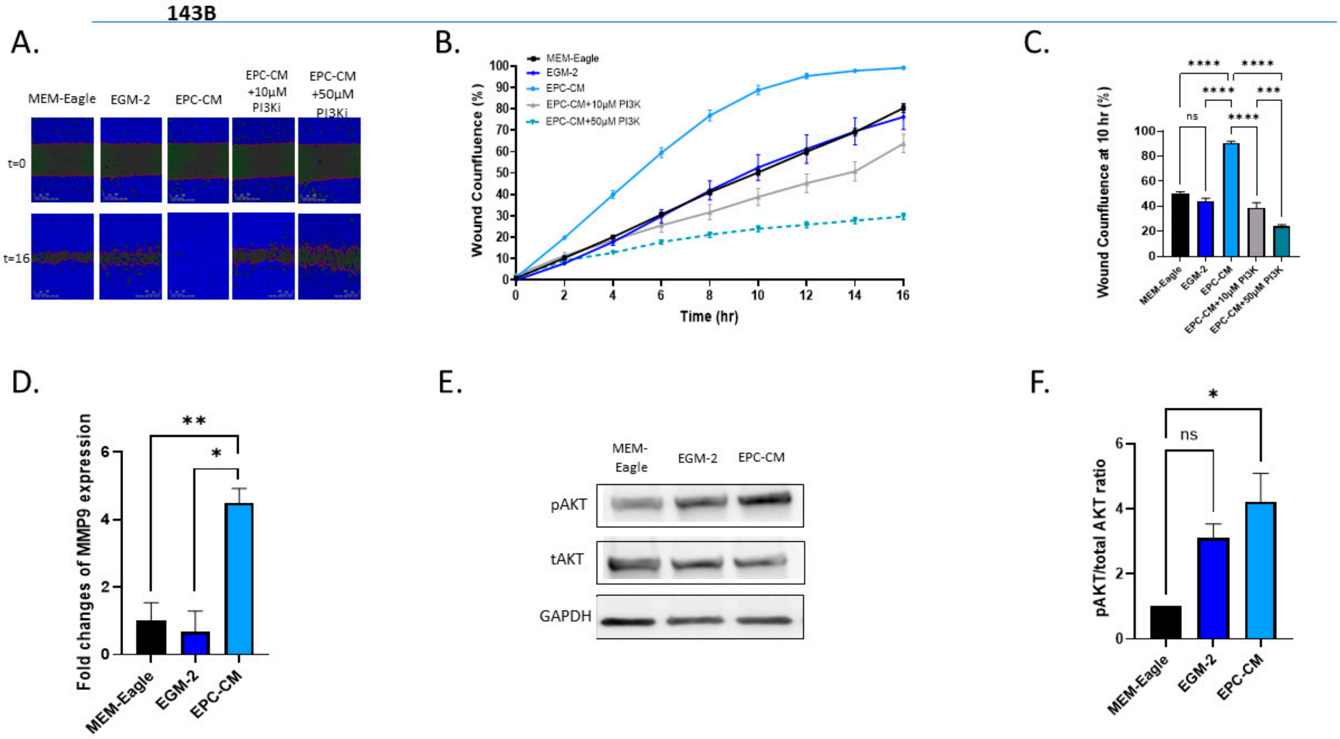

Figure S2: EPCs promote OS migration and invasion via PI3K/AKT signaling. **(A)** Representative time-lapse microscopy images of 143B cells under different culture conditions: MEM-Eagle, EGM-2, EPC-CM, EPC-CM+10μM PI3Ki and EPC-CM+50μM PI3Ki at 0 and 16h. Images were obtained under x10 magnification. Scale bar denotes 100μm. **(B)** 143B cell migration rate. **(C)** Statistical analysis of 143B wound confluence under different culture conditions at 10h. Wound confluence of cells cultured with EPC-CM was significantly higher compared to both controls (MEM-Eagle and EGM-2), **** $p<0.0001$, ns=not significant. Addition of PI3Ki to EPC-CM significantly attenuated OS cell migration in a dose-dependent manner compared to EPC-CM alone. ***$p<0.001$, ****$p<0.0001$. **(D)** Real-time PCR analysis. 143B cells were incubated in MEM-Eagle, EGM-2 or EPC-CM. Real-time PCR results revealed higher MMP9 expression level in the EPC-CM group compared to the controls, *$p<0.05$, ** $p<0.01$. **(L)** Western blot analysis images. 143B cells were incubated in MEM-Eagle, EGM-2 or EPC-CM. Phosphorylated AKT/total AKT and GAPDH antibodies were used to determine the pAKT/total AKT ratios. **(J)** Quantitative analysis of pAKT/tAKT ratios. EPC-CM significantly enhanced AKT phosphorylation in 143B cells compared to MEM-Eagle, *$p<0.05$, ns=not significant.

EPC-CM did not affect U2-OS cell proliferation.

The scratch assay examines cell proliferation and migration abilities. EPC-CM enhanced U2-OS wound closure in the scratch assay. To investigate whether EPC-CM affects both proliferation and migration of U2-OS cells, we conducted an XTT cell proliferation assay (Biological Industries Ltd., Beit-Haemek, Israel). Cells were seeded at a density of 5,000 cells/well in 96-well plates (5 independent experiments). Following an overnight incubation, cells were cultured in : Low DMEM- control or EPC-CM (1:1 with low DMEM). The reaction solution was prepared from activation solution and XTT reagent (Biological Industries Ltd., Beit-Haemek, Israel) in 0.1:5 ratio. 50 μl of reaction solution were added to each well and the plates were then incubated in a standard 5% CO_2 incubator at 37°C. Absorbance was measured by a spectrophotometer at 475nm, 2 h after XTT addition. Net absorbance was calculated by subtracting the absorbance at 475 from the absorbance obtained at 660 nm (to

eliminate the background). EPC-CM did not affect U2-OS cell proliferation (Fig S1C); we therefore conclude that EPC-CM enhances only the migration of U2-OS cells.

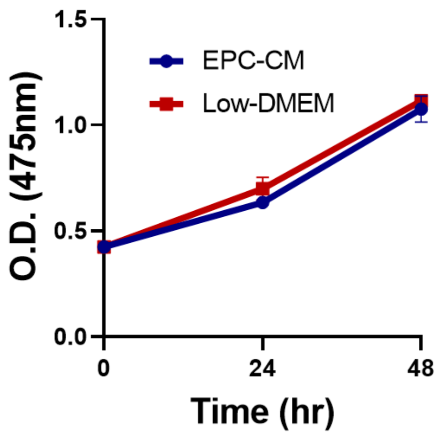

Figure S3: EPC-CM did not enhance U2-OS cell proliferation rate. U2-OS cells were cultured in EPC-CM or Low DMEM (control). EPC-CM did not affect U2-OS cell proliferation when compared to Low-DMEM ($p>0.05$).

VEGF-A and FGF2 promote MMP9 gene expression in OS cells

Mass spectrometry proteomics analysis of EPC-CM revealed a *bona fide* angiogenic profile (Fig 4 A). Among the proteins that were identified in the EPC-CM were: CCL-2, PDGF, SDF-1, PDGF, VEGF-A, EGF, FGF2 and IGF1. To identify which of these growth factors (GFs) increased OS MMP9 gene expression, different combinations of GFs were exogenously added to U2-OS cells for 48h, and MMP9 gene expression was determined in each group. The concentrations of the growth factors are presented in figure S3 A. A combination of GFs containing VEGF-A, FGF2, IGF and EGF enhanced U2-OS MMP9 gene expression by 3.4-fold compared to EGM-2 alone ($p<0.01$). However, a combination of GFs containing IGF, EGF, SDF-1, PLGF and CCL2 did not alter MMP9 gene expression. Remarkably, addition of VEGF-A + FGF2 enhanced U2-OS MMP9 gene expression by 3.2-fold. Therefore, in the following experiments we focused on the combination of VEGF-A and FGF2. In addition, the PI3K inhibitor significantly decreased OS MMP9 expression compared to VEGF-A+FGF2 alone (Fig S4 B).

A

IGF	10ng/ml
EGF	3µg/ml
SDF-1	50ng/µl
PDGF	20ng/µl
CCL2	10ng/µl
VEGF-A	250pg/ml
FGF2	1µg/ml

B

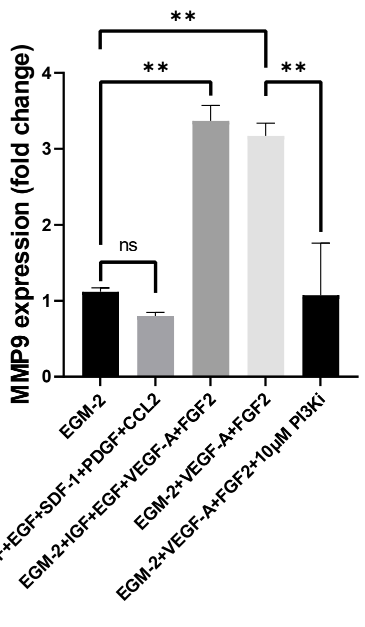

Figure S4: The combination of VEGF-A and FGF2 promotes MMP9 gene expression in OS cells as revealed by real-time PCR analysis. U2-OS cells were incubated in: (1) EGM-2 (without supplements), (2) EGM-2+IGF+EGF+SDF-1+PDGF+CCL2, (3) EGM-2+VEGF-A+FGF2+IGF+EGF, (4) EGM + VEGF-A + FGF2 and (5) EGM + VEGF-A + FGF2+10µM PI3Ki. (A) Table of growth factors concentrations added to EGM-2. (B) Real-time PCR results revealed a markedly increased MMP9 gene expression in OS cells that were incubated with a combination of VEGF-A + FGF-2 (** $p<0.01$). Other growth factors did not enhance MMP9 gene expression.

IgG antibody did not affect OS migration compared to EPC-CM.

Normal goat IgG antibody was used as a negative control for the scratch assay and was added to EPC-CM. This IgG antibody did not affect U2-OS migration when compared to EPC-CM (Fig S1B).

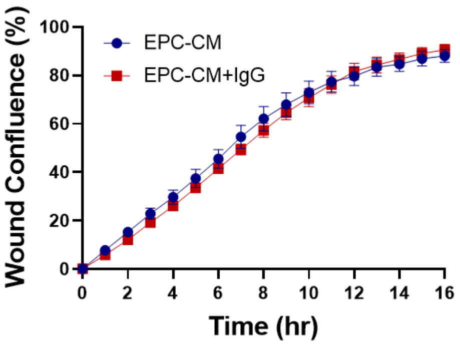

Figure S5: Migration rate of U2-OS cultured in: EPC-CM / EPC-CM+IgG. The addition of the IgG antibody to EPC-CM, did not affect U2-OS cell migration, compared to EPC-CM alone ($p>0.05$).

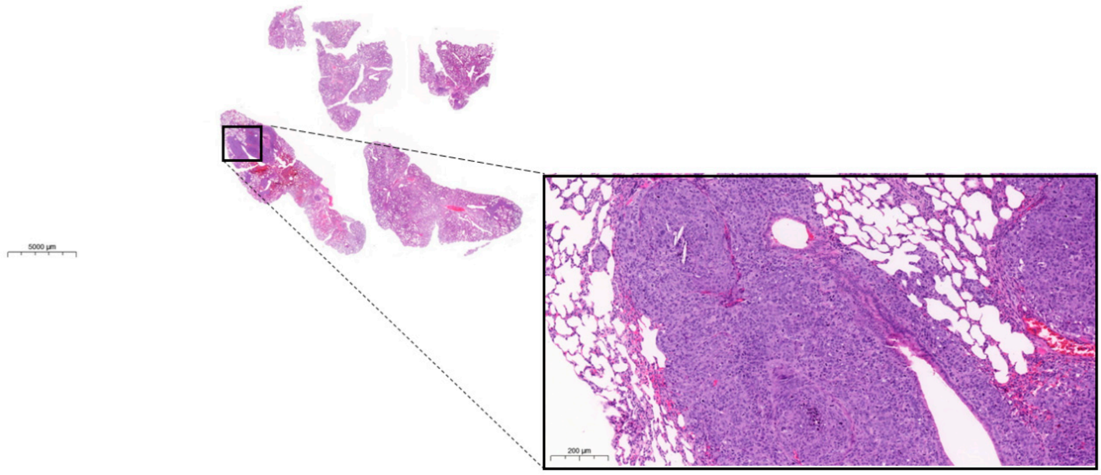

Figure S6: Representative image of 143B lung metastasis at 5 weeks. Images were obtained under x0.5 and x10 magnification. Scale bar denote 5000 µm and 200 µm .

Endothelial progenitor cells secretome analysis

Table S2: Accession number of proteins identified in EPCs secretome.

E9PL09
F8VWS0
B4DXW1
P04083
P07355
P61769
B1AK87
P31944
P07858
E9PNW4
P29279
P01034
P21291
P53634
Q12805
P26641
Q9NQ30

Q12841
P09382
P06744
P09211
A0A0C4DGL8
Q16270
P03956
A0A0A0MTC7
P26022
P05121
P02545
P25787
O00622
H7BZJ3
P30101
P30613
H3BMH2
Q92743
P09486
Q99536
Q8NBS9
G3V1Q4
P50552
P04424
P26038
F8VPF3
P61812
Q01995
B4DLR8
E2QRB9
O15143
P05090
P22626
Q5H9A7
P23284
B1ALD9
P28066
Q5JR08
O95084
P13489
F8WE86
F6RFD5
G3V576
P61457
Q99832
P04066
P23381
O43175
P37837
P59998
P45877
A0A087WWU8

Q5VU61
P31946
P62258
Q04917
P61981
P31947
P27348
P63104
J3QQM1
F8VVW8
P62851
P62857
M0R0F0
A0A0B4J1R4
P49189
P30050
P52209
P11021
Q9BWD1
E9PLJ2
P68032
P60709
O15144
P55263
P23526
A0A096LNY6
Q01518
P61204
P18085
P14550
P11766
H0Y804
B4DQI4
Q10469
C9JV77
P02765
P01023
P04745
P06733
P02771
P01008
P06727
P02649
Q5SRP5
P00966
Q9BXN1
P98160
P15291
O43505
P02749
Q562R1
P13929

Q93088
K7ESE8
Q96CX2
A0A087X0K1
A0A087X271
P27797
P00918
P16152
P15169
G3XAP6
P04040
A0A1B0GW44
Q9UBR2
P60953
Q9NTU7
E9PFZ2
O00299
Q9Y696
Q14019
P00740
P12259
P00742
G3V1A4
P02452
P02458
P02461
P20849
A0A087WXW9
P12109
P27658
P12107
D6RGG3
H7C457
F5GZK2
A0A087WTA8
P08572
Q96CG8
Q9BXJ4
Q9BXJ0
P01024
A0A140TA32
P01031
F5GY80
P02748
P00751
P00746
A0A087X0I2
P31146
Q9ULV4
Q9Y240
D6RF92
C9J0E4

Q15828
C9JFR7
P28838
Q9H7Y0
P13716
Q08554
Q14117
P63167
Q14118
O95834
E9PRU1
Q504U8
P68104
P13639
Q9UNN8
P60842
E7EQG2
P05198
I3L397
P52907
C9JUG7
Q16658
Q8IY13
P02671
P02675
C9JEU5
A0A1B0GVK0
Q06828
P02751
P23142
Q5HY54
Q5JXI8
P09467
P04075
P05062
P09972
P16930
P17931
P47929
Q92820
J3KRG2
Q5T0I0
A0A0A0MS51
B5MCZ9
A0A087X1J7
P08263
Q16772
Q7RTV2
E9PLF1
P04406
O14556
Q5VZR0

P62826
P62873
E7EP32
D6RF23
G3V1N2
P48723
P0DMV9
E9PKE3
P04792
P07900
P08238
P69905
E9PFT6
E9PBW4
P05546
Q04756
P26927
D6R9P3
D6RF44
P16403
Q96KK5
P06899
O60814
K7EMV3
P62805
A0A140T8W8
Q93099
Q86YZ3
F6VDH7
A6XND0
P01344
P18065
Q13418
Q5T985
A0A087WW43
Q14624
O75874
P14923
E7EQB2
Q8TF66
P00338
P07195
J3KS22
P10619
P61626
Q14764
P40925
O15232
P16035
P20774
P28482
Q99972

B4DYP1
O95865
Q96PD5
P48163
C9JKZ2
E9PKG6
Q13232
P15531
P22392
F8VRJ2
Q9NRN5
O95497
P62937
A0A0A0MSI0
P32119
H7C3T4
P30041
P30086
P36871
P00558
P18669
Q9Y617
P36955
Q13835
C9JCT1
Q9BX97
P00747
P13796
Q15365
H3BRU6
Q6NZI2
A6NLN1
F5H6Q2
P0CG39
P20742
Q8N0Y7
Q15113
P07737
P12273
Q06323
P25786
P25788
H0YN18
P60900
O14818
P20618
A0A087WVV1
K7ELW0
Q15084
P07237
Q9NUQ9
G5E9F8

P31949
P05109
P06702
Q08188
P22735
A0A0U1RR20
P00734
P00491
P14618
Q08257
P31150
P50395
Q15404
P63000
B1AH77
P61026
P62820
Q9H0U4
P51148
P61006
P61224
Q15493
Q15293
Q96D15
F8W914
P00352
Q5VY30
J3KTF8
F5H3P3
P34096
H0YBX3
Q00266
P31153
D6RDU5
Q9NQ38
C9J9S3
C9J9C1
P62714
P02787
P50454
A0A0C4DGB6
P02743
Q5T123
Q5T750
B1AN48
Q9H2M3
H0YLA4
H0YB13
Q6UWP8
Q9Y490
Q9Y4G6
P50991

P50990
E9PHK0
A6NNI4
P10599
P07996
P05543
C9JBB3
G3V0E5
Q03167
Q15582
P37802
J3KPG2
P60174
H7BYY1
P67936
P07477
Q9BQE3
P68366
P07437
Q9H4B7
Q13885
P68371
Q9BUF5
Q13404
O60701
A0A087WYS1
Q12907
P08670
P02774
P04004
Q5VU13
O75083
P12955
